# Case Report: Atypical Solitary Brain Metastasis: The Role of MR Spectroscopy In Differential Diagnosis

**DOI:** 10.3389/fonc.2022.866622

**Published:** 2022-07-22

**Authors:** Dusko Kozic, Nebojsa Lasica, Danica Grujicic, Savo Raicevic, Natasa Prvulovic Bunovic, Igor Nosek, Jasmina Boban

**Affiliations:** ^1^ Faculty of Medicine, University of Novi Sad, Novi Sad, Serbia; ^2^ Oncology Institute of Vojvodina, Center for Diagnostic Imaging, Sremska Kamenica, Serbia; ^3^ Clinic of Neurosurgery, Clinical Center of Vojvodina, Novi Sad, Serbia; ^4^ Faculty of Medicine, University of Belgrade, Belgrade, Serbia; ^5^ Neuro-Oncology Department, Clinic of Neurosurgery Clinical Center of Serbia, Belgrade, Serbia; ^6^ Pediatric Oncology Department, Institute of Oncology and Radiology of Serbia, Belgrade, Serbia; ^7^ Department of Pathology, Clinical Center of Serbia, Belgrade, Serbia

**Keywords:** brain metastasis, nonenhancing, magnetic resonance spectroscopy, case report, neuroendocrine tumor

## Abstract

**Background:**

Metastatic brain tumors are typically located at the cerebral hemispheres or the cerebellum and most frequently originate from primary breast or lung tumors. Metastatic lesions are usually associated with blood–brain barrier disruption, solid or ring-like contrast enhancement, and perilesional vasogenic edema on brain imaging. Even in cases where metastases are predominantly cystic, enhancement of the minor solid component can be detected. In contrast, non-enhancing secondary brain tumors were only reported in a patient after antiangiogenic treatment with bevacizumab.

**Case report:**

We report a case of a 54-year-old male who presented with left-sided weakness and multiple seizures. Brain magnetic resonance imaging revealed a T2-weighted heterogeneous solid tumor in the right frontoparietal parasagittal region, with no apparent enhancement on T1-weighted post-contrast images and no evident perilesional edema. Further MRS analysis revealed markedly increased choline and lipid peaks. The patient underwent craniotomy for tumor removal. Histopathology revealed findings consistent with metastatic non-microcellular neuroendocrine lung cancer. positron emission tomography/computed tomography (PET/CT) revealed a stellate lesion within the right upper lung lobe, compatible with primary lung cancer.

**Conclusion:**

Non-enhancing brain metastatic tumors are rarely reported in the literature, usually following antiangiogenic treatment. Here, we report the first ever case of a non-enhancing metastatic brain tumor with no prior history of antiangiogenic treatment, with particular emphasis on the importance of MRS analysis in atypical brain lesions.

## Background

Magnetic resonance imaging (MRI) is the method of choice for detection of brain metastases (1). Majority of metastatic brain tumors are located in the cerebral hemispheres and the cerebellum, whereas breast and lungs are the most frequent primary origin of the neoplastic dissemination (1, 2). Metastatic foci are associated with blood–brain barrier disruption, solid or ring-like contrast enhancement, and perilesional vasogenic edema are usually evident. Even when cerebral metastatic foci are predominantly cystic, enhancement of the minor solid component can be detected. Non-enhancing solid components of brain neoplasms are usually evident in World Health Organization (WHO) grade II and grade III astrocytomas but seldom in metastatic lesions (3–5). Karimi et al. reported non-enhancing metastases in a patient with lung cancer, following antiangiogenic effects of bevacizumab (Avastin) (5).

To the best of our knowledge, this is the first report of a non-enhancing solid component of metastatic brain tumor and the benefits and importance of the magnetic resonance spectroscopy (MRS) as a supportive non-invasive diagnostic tool in the diagnostic algorithm.

## Case presentation

A 54-year-old male presented with a 4-week history of the left arm and leg weakness and clumsiness followed by multiple seizures 2 weeks prior to admission. The patient had no past medical history or history of hereditary diseases. Physical examination was unremarkable. Neurological examination revealed left-sided weakness with no sensory deficits.

**Figure 1:**
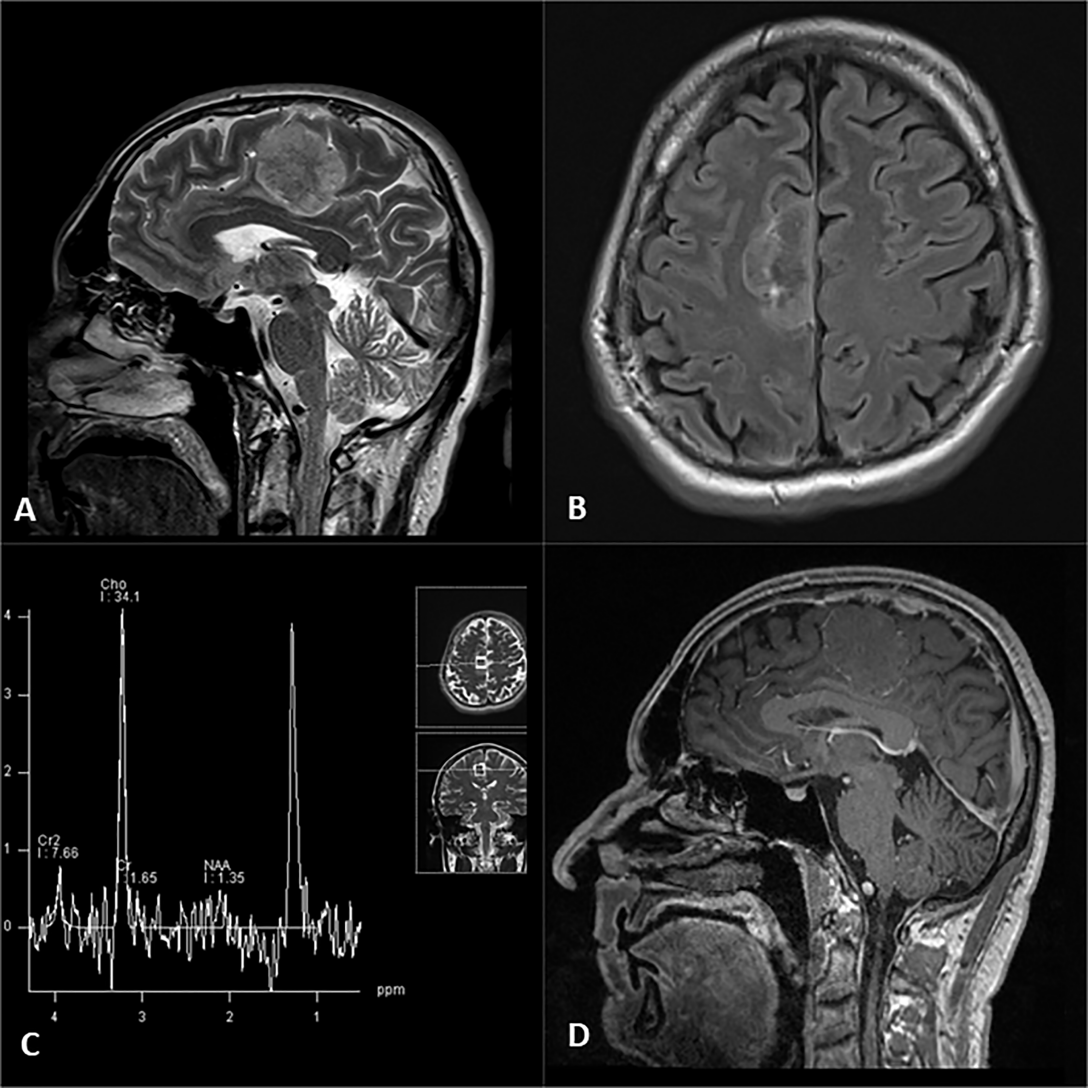
Right frontal parafalcine tumor evident on T2 sagittal **(A)** and FLAIR axial images **(B)**, associated with typical high choline and lipid peaks on single-voxel MR spectroscopy **(C)** and lack of expected enhancement on post contrast T1 sagittal image **(D)**.

Brain MRI revealed a dural-based mass in the right frontoparietal region, extending to the intraaxial compartment, measuring 46 mm in antero-posterior, 34 mm in cranio-caudal, and 23 mm in the lateral dimension. The mass was heterogeneous on the T2-weighted sequence with no significant post-contrast enhancement of the solid tumor component after intravenous administration of gadolinium-diethylenetriamine pentaacetic acid. Tortuous peripheral enhancement correlated with dislocated cortical venous vessels. Markedly increased choline (Cho) and lipid peak were noted on MRS (**Figures 1A–D**). No surrounding edema was noted. Imaging findings mainly were consistent with a solitary fibrous tumor.

After usual prepping of the operative field, right parasagittal craniotomy was performed for tumor resection. The lesion was in the right parietal parasagittal region in contact with falx, pink in color, with an approximate largest diameter of 3.5 cm. Gross total resection was performed with the assistance of neuromonitoring. Tissue samples were taken intraoperatively and sent for frozen section and histopathology examination. The patient postoperative course was uneventful.

**Figure 2:**
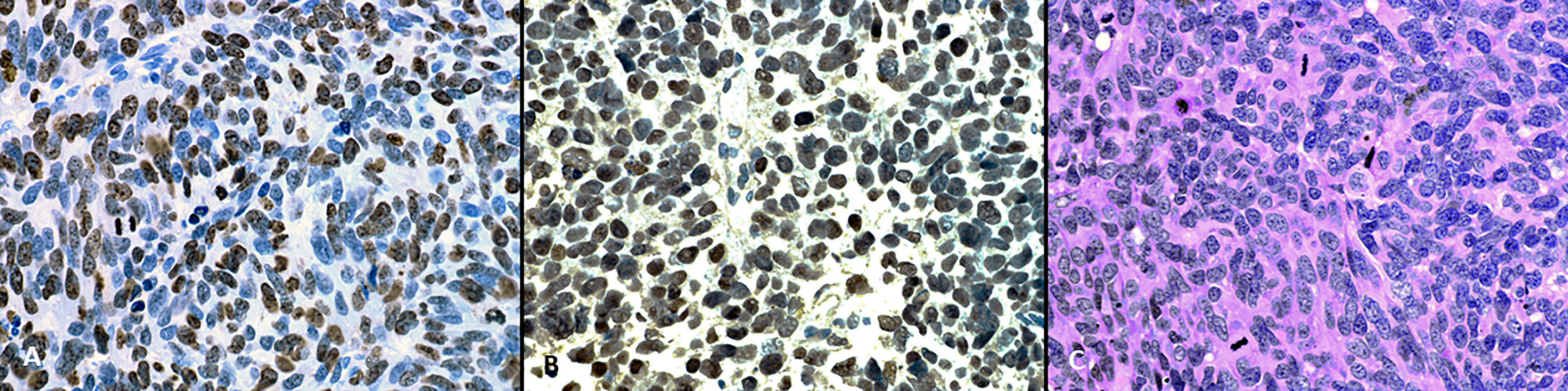
**(A)** On hematoxylin and eosin staining tumor cells have scant to moderate amount of eosinophillic cytoplasm, and irregular nuclei with “salt and pepper” chromatin. Brisk mitotic activity can be seen. **(B)** On immunohistochemical staining tumor cells nuclei strongly and diffusely express TTF-1. **(C)** The proliferative Ki-67 index is high (magnification, x400).

**Figure 3:**
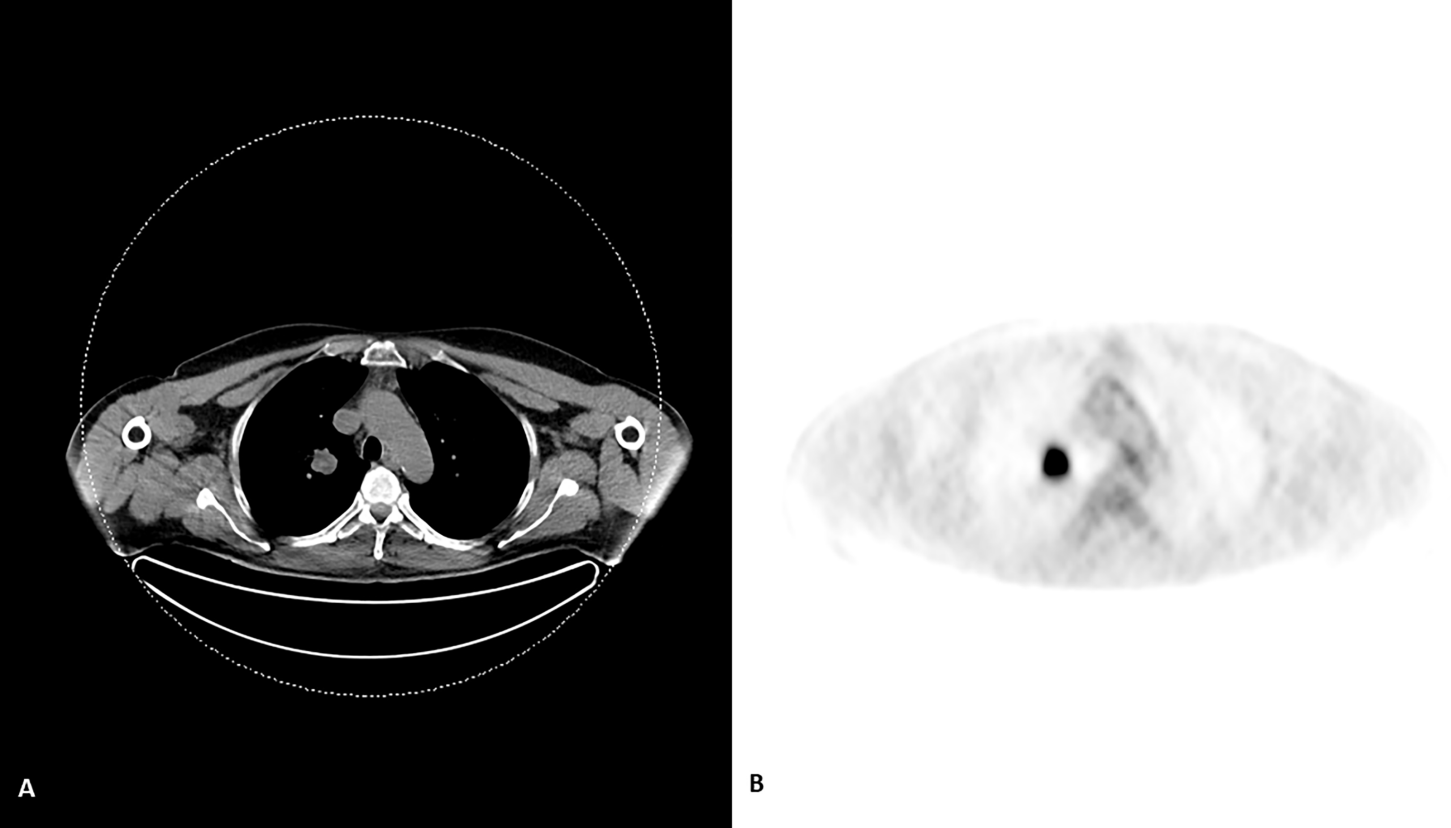
An evident lesion in the upper lobe of the right lung on CT scan **(A)** with an avid FDG uptake **(B)** of the compatible with cancer on PET scan.

On histopathological examination tumor cells manifested scant to moderate amounts of eosinophilic cytoplasm and irregular nuclei with “salt and pepper” chromatin. Brisk mitotic activity was noted (**Figure 1A**). Immunohistochemical staining revealed that tumor cells nuclei strongly and diffusely expressed TTF-1 (**Figure 1B**) associated with high proliferative Ki-67 index (**Figure 2C**). These features were compatible with non-microcellular neuroendocrine lung cancer metastatic disease. PET/CT revealed a stellate lesion, with spicules radiating out from the central portion of the mass, within the right upper lung lobe, compatible with primary lung cancer, measuring 17 mm × 2 8mm (**Figure 3**).

Patient remained progression free for 2 years, developing multiple metastases in cerebral and cerebellar hemispheres, lungs, and adrenal glands. Further institutional protocol-based adjuvant therapy included etoposide with cisplatin chemotherapy (EP) and radiation treatment for the tumoral bed with fractioned external beam irradiation to a total dose of 40 Gy divided into 20 fractions, which was interrupted after 17th fraction due to patient’s personal will. Extensive dissemination of malignant disease resulted in lethal outcome three years after the initial diagnosis had been established. 

## Discussion

Non-enhancing cerebral metastatic foci are seldomly reported and poorly understood. Typically, metastatic lesions to the central nervous system demonstrate a strong contrast enhancement and are detected on post-contrast T1-weighted images, whereas plain and post-contrast FLAIR sequences are found to be most sensitive in identifying leptomeningeal secondary malignant involvement (6, 7). However, occasionally leptomeningeal metastases may exhibit minimal or even absent contrast enhancement, particularly if there is a presence of tumor hemorrhage as reported earlier by Rumboldt et al. (8); these lesions, however, usually show precontrast T1W hyperintensity, which allows for better initial detection. Early detection of brain metastases may improve disease control and life expectancy due to prompt implementation of adequate treatment (9, 10).

Neovascularization of the metastatic lesions and hyperemia is a major cause of contrast enhancement, associated with blood volume and blood flow increase. An additional contributing factor in metastatic tumors enhancement is compatible with alterations in blood–brain barrier because blood vessels supplying the tumor tissue are typically immature and “leaky” (11–13).

The most likely reason for the non-enhancement of cerebral metastases, documented in the literature, is associated with the antiangiogenic effects of anticancer treatment and monoclonal antibodies (5, 14). Another reason is associated with delayed or even completely blocked permeability of the blood–brain barrier to gadolinium chelate due to tiny fenestrations, diminished local blood flow, and the size capillary surface area (15, 16).

However, our patient presented with no clinical history of oncologic disease or anticancer, biological, or immunosuppressive treatment. The tumor on non-enhanced MRI study was rather voluminous, most compatible with meningioma, or less likely, metastatic neoplastic process. Primary brain tumor, with the secondary invasion of the dura, was also considered. The absence of expected vivid contrast enhancement practically excluded meningioma and metastatic lesion from the differential diagnosis. However, the neurometabolic profile revealed on MRS noted high peaks of Cho and lipid and no identification of N-acetyl aspartate (NAA) and creatine (Cr). Such a pattern is most compatible with metastasis.

MRS is a non-invasive diagnostic modality that evaluates the cerebral metabolic profile by determining different neurochemical concentrations. NAA, seen at 2.06 ppm in the proton spectrum of a healthy person, is a marker of neuronal integrity and function. The increased Cho concentration indicates the presence of increased cell membrane turnover. The elevated lactate peak is associated with increased macrophage activation and anaerobic glycolysis. The level of Cr seems to be related to cerebral energy reserves. Recent studies suggested that the determination of absolute concentrations of each metabolite substantially increases the accuracy and understanding of the altered brain physiology (17). In almost all patients with brain tumors, MRS shows metabolic ratio alterations (18).

The decreased level of NAA is consistent with the destroyed neuronal tissue, neuronal dysfunction, or rarefaction. The increased Cho peak in primary and metastatic brain tumors is associated with accelerated cell proliferation and cell membrane turnover (19). The rise of the myo-inositol peak in the neoplastic tissue is most compatible with glial proliferation in less aggressive gliomas (grade 2), whereas complete lack of NAA and Cr peaks may suggest a non-glial origin of the neoplasm (20).

Elevated Cho and lipid peaks, with absent peaks of NAA and Cr, are typical of metastatic disease of the brain, whereas a Cho-only spectrum is characteristic of non-glial intra-axial neoplasms and extra-axial tumors. The additional presence of alanine is seen in meningiomas (21). This pattern, characteristic for metastatic brain tumors, was evident in our patient, increasing the role of MR spectroscopy, especially in atypical appearances of different cerebral neoplastic diseases of glial and non-glial nature.

## Conclusion

In previous studies, non-enhancing brain metastatic tumors were only reported following antiangiogenic treatment. To best of our knowledge, this is the first ever case of a non-enhancing solid component of metastatic brain tumor with no prior history of antiangiogenic treatment, with particular emphasis on the importance of MRS analysis in atypical brain lesions.

## Data Availability Statement

The raw data supporting the conclusions of this article will be made available by the authors, without undue reservation.

## Ethics Statement

The studies involving human participants were reviewed and approved by Ethical Review Board of the Clinical Center Serbia. The patients/participants provided their written informed consent to participate in this study. Written informed consent was obtained from the individual(s) for the publication of any potentially identifiable images or data included in this article.

## Author Contributions

DK, DG, and NL wrote the manuscript. SR undertook the pathological analysis of the specimens. DK, NPB and IN undertook the radiological diagnosis. DG carried out surgical and the clinical management of the patient. JB was involved in data analysis and critical revision of the drafted manuscript. All authors contributed to the article and approved the submitted version.

## Conflict of Interest

The authors declare that the research was conducted in the absence of any commercial or financial relationships that could be construed as a potential conflict of interest.

## Publisher’s Note

All claims expressed in this article are solely those of the authors and do not necessarily represent those of their affiliated organizations, or those of the publisher, the editors and the reviewers. Any product that may be evaluated in this article, or claim that may be made by its manufacturer, is not guaranteed or endorsed by the publisher.
